# DIDAF-Depth: Dual-Path Interaction and Dual-Attention Fusion Network for Self-Supervised Nighttime Monocular Depth Estimation

**DOI:** 10.3390/jimaging12080362

**Published:** 2026-08-07

**Authors:** Qing Chen, Chao Wei, Bingmeng Zhu, Qiang Yan, Shengbing Chen, Dongmei Zhou

**Affiliations:** 1 Tracy (Chengdu) Information Technology Co., Ltd., Chengdu 610041, China; tracy20120510@sina.com (Q.C.); yanqiang1987315@163.com (Q.Y.); 2 School of Mechanical and Electrical Engineering, Chengdu University of Technology, Chengdu 610059, China; weichao@stu.cdut.edu.cn (C.W.); 2023051269@stu.cdut.edu.cn (S.C.); zhoudm@cdut.edu.cn (D.Z.)

**Keywords:** nighttime monocular depth estimation, self-supervised learning, Transformer-CNN interaction, attentional feature fusion

## Abstract

Self-supervised monocular depth estimation is challenging at night because adverse illumination degrades the visual cues required for correspondence estimation and depth inference. Although appearance compensation and domain transfer can mitigate nighttime visual variations, reliable depth recovery under such conditions still depends on exploiting incomplete local structural cues and uncertain scene context. To address this challenge, we propose the Dual-Path Interaction and Dual-Attention Fusion Network (DIDAF-Depth), which improves nighttime depth recovery through coordinated convolutional neural network (CNN)–Transformer interaction, attentional feature fusion, and structure-preserving reconstruction. Specifically, we design the Transformer-CNN Vertical Interaction Fusion (TC-VIF) encoder to perform bidirectional cross-layer exchange, allowing local structural cues and global scene context to complement and progressively refine one another during feature extraction. We further develop the Dual-Coupled Attentional Fusion Module (DCAFM) to model spatial and channel interdependencies and selectively integrate complementary local and global information into a unified representation for depth decoding. Building on DCAFM’s unified representation, we construct the Edge-aware Densely Cascaded Multi-scale Network (EDCMN) to propagate features across scales, reinforce weak boundaries during upsampling, and preserve structural continuity in predicted depth maps. Experiments on the nighttime subsets of the Oxford RobotCar and nuScenes datasets indicate that DIDAF-Depth provides strong and consistent performance under the adopted evaluation protocols, supporting the effectiveness of the proposed framework.

## 1. Introduction

Radar and light detection and ranging (LiDAR) sensors provide direct range measurements, but they require additional hardware and calibration [1,2]. Monocular depth estimation instead infers dense depth from camera images, providing a camera-only option for robotics and navigation [3], autonomous driving perception [4], and direct monocular depth estimation [5,6]. In recent years, supervised methods have achieved substantial progress [5,6,7], but their dependence on large-scale depth annotations entails costly data acquisition and makes performance sensitive to LiDAR or time-of-flight measurement quality. Self-supervised approaches instead derive supervisory signals from geometric consistency across stereo pairs or monocular video [8,9,10,11]. Most advances and evaluations, however, remain centered on daytime benchmarks such as KITTI [12], Cityscapes [13], and DDAD [14], where visual correspondence is comparatively reliable.

**Figure 1:**
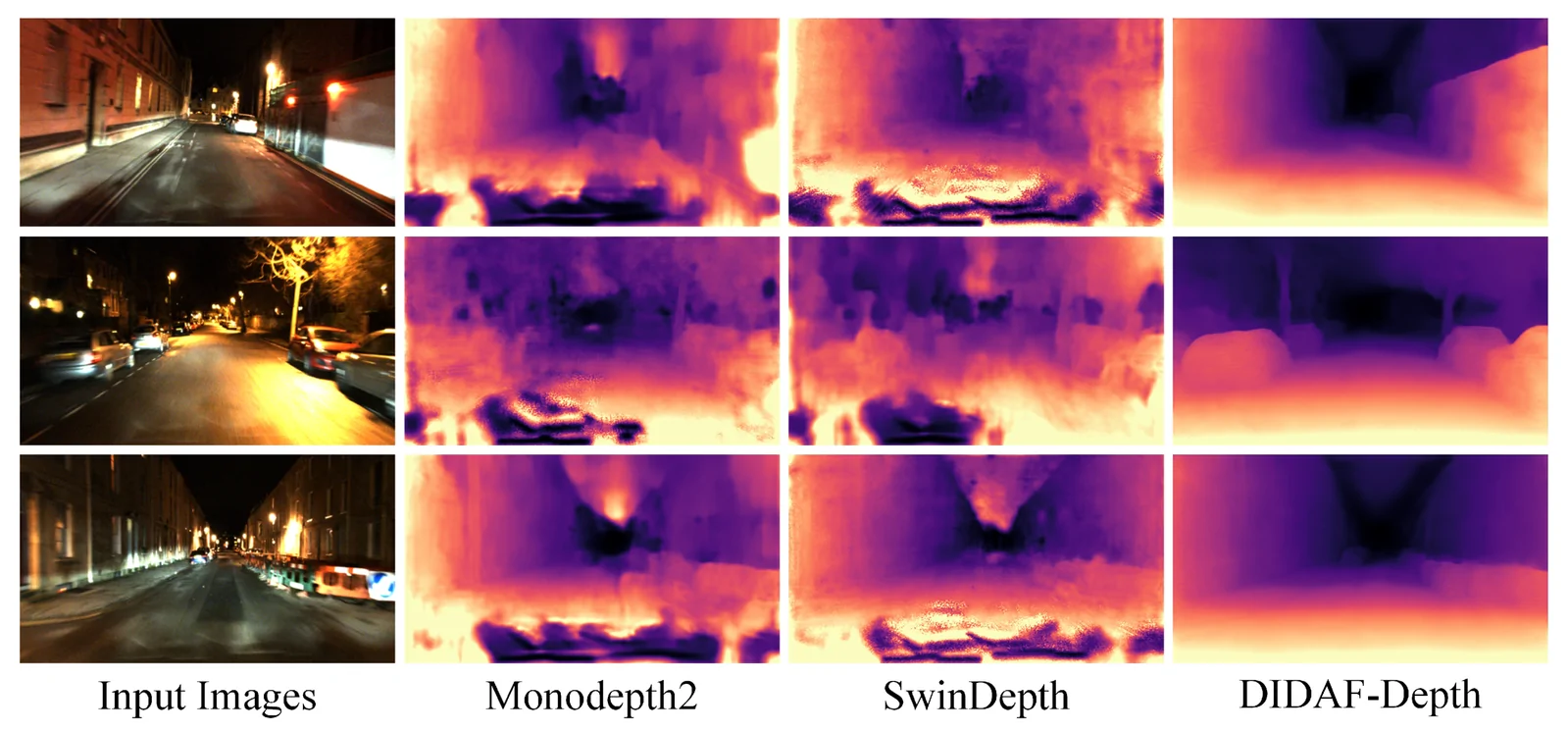
Failure of daytime-oriented depth-estimation methods under low and spatially non-uniform nighttime illumination. Columns show RobotCar-Night inputs and predictions from Monodepth2 [15], SwinDepth [16], and the proposed method.

Nighttime scenes violate this favorable condition in several coupled ways. Low illumination and local overexposure suppress texture contrast and blur object boundaries, weakening the visual correspondences required for depth estimation. As illustrated in Figure 1, representative daytime architectures produce structurally inconsistent nighttime predictions. The CNN-based Monodepth2 suffers local depth distortions when evidence is sparse, whereas the Transformer-based SwinDepth over-smooths weak boundaries despite retaining coarse global structure. This contrast makes CNN–Transformer cooperation attractive, but it also exposes a more fundamental challenge. Their heterogeneous representations must remain mutually informative despite illumination-dependent corruption.

Existing nighttime approaches mainly improve image appearance, reduce domain discrepancy, or suppress unreliable photometric supervision. Although these interventions increase observation reliability, the depth network must still infer geometry from incomplete visual evidence. The remaining challenge is therefore architectural: how to preserve and coordinate complementary information as degraded observations are transformed into depth predictions.

To address this challenge, we propose the Dual-Path Interaction and Dual-Attention Fusion Network (DIDAF-Depth), a self-supervised method for nighttime monocular depth estimation. The main contributions are as follows:

- We design the Transformer-CNN Vertical Interaction Fusion (TC-VIF) encoder, which inserts ordered bidirectional exchange between adjacent encoding stages. By allowing CNN-derived local structure and Transformer-derived scene context to refine one another while features are formed, the TC-VIF encoder produces complementary representations from incomplete nighttime evidence.
- We propose the Dual-Coupled Attentional Fusion Module (DCAFM) to address the residual mismatch between these interacting representations. Its coupled spatial and channel attention pathways model cross-branch dependencies and selectively consolidate complementary responses into a unified representation for depth decoding.
- For depth reconstruction, we construct the Edge-aware Densely Cascaded Multi-scale Network (EDCMN) as a structure-aware decoder. Its multi-scale propagation and dense connections maintain information flow across decoding stages, while edge-aware upsampling reinforces weak boundaries during resolution recovery.
- Extensive experiments and ablation studies on RobotCar-Night and nuScenes-Night validate the proposed components and demonstrate robust depth estimation across different scene ranges and evaluation settings.

## 2. Related Work

### 2.1. Transformer-CNN in Depth Estimation

**Figure 2:**
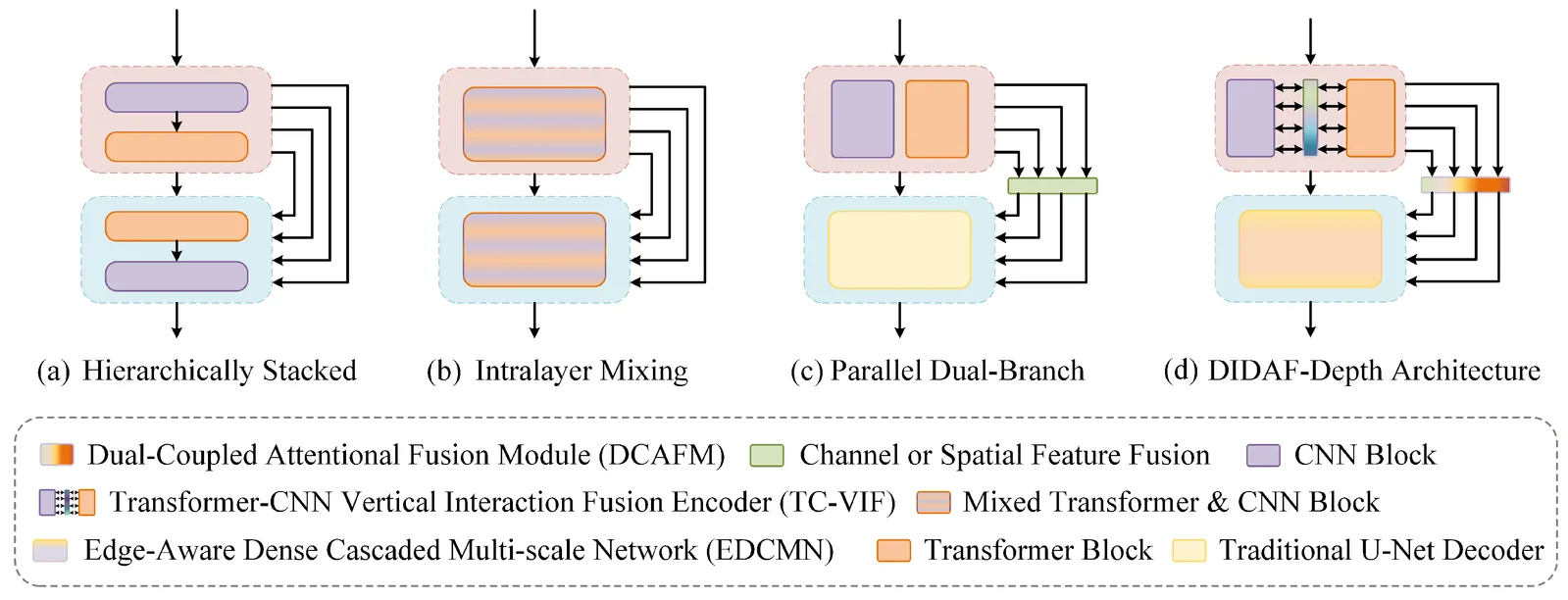
Representative Transformer–convolutional neural network (CNN) architectures for depth estimation: (**a**) hierarchical stacking, (**b**) intralayer mixing, (**c**) parallel dual branches with lateral feature fusion, and (**d**) the proposed Dual-Path Interaction and Dual-Attention Fusion Network (DIDAF-Depth). The proposed architecture combines vertical bidirectional interaction, dual-coupled attentional fusion, and edge-aware multi-scale decoding.

Hybrid Transformer-CNN depth encoders exploit complementary inductive biases: CNNs retain local geometric detail, whereas Transformers capture long-range scene relationships [17,18,19,20]. How these inductive biases are coordinated determines the extent to which local structure and global scene context can refine one another as features are formed. Figure 2 illustrates the representative information-flow patterns used to establish this coordination.

These architectures differ primarily in how they organize the CNN and Transformer pathways and route information between them across encoding stages. Staged designs, such as that of Hwang et al. [17], form local convolutional features before introducing Transformer-based global reasoning. This simple hierarchy makes information flow largely one-way, so later context cannot revise local representations formed in earlier stages. Within-stage hybrids such as MonoViT [18] shorten this delay by interleaving convolution and attention at each level. Their tighter coupling supports immediate exchange, but confines both operations to a shared pathway and matched scale. Parallel architectures such as GlocalFuse-Depth [19] and TDCNet [20] instead preserve branch-specific representations and combine them through stage-matched lateral fusion. Although this separation protects their distinct inductive biases, interaction remains horizontal and intermittent, allowing discrepancies to accumulate as the branches deepen.

This divergence is amplified at night, where low illumination and sparse textures weaken local convolutional responses, while glare and noise introduce uncertainty into long-range scene modeling. As shown in Figure 2d, the TC-VIF encoder in DIDAF-Depth couples independent CNN and Transformer pathways through ordered bidirectional exchange between adjacent stages. By carrying the exchanged features forward in both branches, the encoder allows local structural cues to constrain uncertain global responses and global context to supplement weakened local evidence throughout encoding. The resulting representations remain complementary yet progressively coordinated, providing a more reliable basis for subsequent attentional fusion and structure-preserving depth reconstruction.

### 2.2. Nighttime Depth Estimation

Nighttime depth methods can be distinguished by where they intervene in the estimation pipeline. One line acts upstream of depth prediction by narrowing the day–night domain gap, allowing geometric knowledge learned from well-lit scenes to remain useful. Across adversarial feature alignment in ADFA [21], frequency-domain adaptation in DNA-Depth [22], and physically guided distribution compensation by Yang et al. [23], the common objective is to make nighttime observations more compatible with daytime depth priors. Domain compensation, however, cannot recover evidence removed by darkness or saturation. Translation-based variants may also disturb the pixel correspondence required for photometric supervision.

A second line intervenes within the depth-learning process, treating nighttime corruption as uncertainty that must be identified or suppressed during optimization. RNW [24] combines Mapping-Consistent Image Enhancement (MCIE) with Statistics-Based Masking (SBM) to regularize unreliable observations. STEPS [25] couples enhancement and depth estimation through illumination-aware maps and masks, bringing appearance recovery into the self-supervised objective. More recent architectural approaches extend this idea from supervision to representation learning. Hou et al. [26], for example, combine a Dual-Axis Transformer (DAT), an Illumination Compensation PoseNet (ICP), and cross-layer Adaptive Fusion (AFM). This progression improves robustness at different stages, but illumination correction and feature adaptation remain only partially coordinated across the complete depth pipeline.

Despite these advances, nighttime degradation still affects multiple stages of depth estimation, from feature extraction to fusion and reconstruction. Existing methods often improve one part of the pipeline, while the coordination between local structure, global context, and boundary preservation remains insufficient. This motivates DIDAF-Depth to jointly calibrate, fuse, and decode nighttime geometric cues within a unified architecture.

## 3. Materials and Methods

### 3.1. Principles and Basic Framework

Self-supervised monocular depth estimation eliminates the need for ground-truth depth labels. It leverages geometric consistency across multiple views to generate supervisory signals, enabling the joint optimization of depth and pose networks. Specifically, the Depth Network is responsible for predicting the depth maps $D_{t}$ of the target image $I_{t}$, while the Pose Network follows the Monodepth2 PoseNet [15] to estimate the relative poses $T_{t\to t-1}$ and $T_{t\to t+1}$ between the target image $I_{t}$ and the temporally adjacent source frames $I_{t-1}$ and $I_{t+1}$. Given the predicted depth and pose, the pixel-wise correspondence between a point $p_{t}$ in $I_{t}$ and its projection $p_{s}$ in $I_{s}$ can be computed as follows:

(1) $$p_{s}∼KT_{t\to s}D_{t}\left(p_{t}\right)K^{-1}p_{t},$$

where ∼ denotes equivalence, and *K* represents the intrinsic matrix of the camera.

According to Equation (1), we employ a differentiable bilinear sampling operation s(·,·) [27] to reconstruct ${\hat{I}}_{t}$ from $I_{s}$ as follows:

(2) $${\hat{I}}_{t}=s\left(I_{s},p_{s}\right).$$

Thus, the photometric error between ${\hat{I}}_{t}$ and $I_{t}$ serves as the primary supervision signal for training both the depth and pose networks. Following Monodepth2 [15] and recent nighttime self-supervised methods, we compute this term using minimum reprojection across the adjacent source frames and apply auto-masking to suppress pixels that do not provide valid motion-based supervision. Following [8], we integrate $\ell_{1}$ and structural similarity index measure (SSIM) [28] terms into a unified photometric loss $L_{p}$, formulated as follows:

(3) $$L_{p}\left(I_{t},{\hat{I}}_{t}\right)=\alpha \frac{1-\mathrm{SSIM}(I_{t},{\hat{I}}_{t})}{2}+\left(1-\alpha \right){∥I_{t}-{\hat{I}}_{t}∥}_{1},$$

where the weighting factor α is set to 0.85 for all experiments.

Since incorrect depth values may still yield correct correspondences under certain relative poses $T_{t\to s}$, we follow [8] and introduce an edge-aware smoothness loss $L_{s}$ to regularize depth prediction and mitigate depth blurring. $L_{s}$ is formulated as follows:

(4) $$L_{s}=\left|\partial_{x}d_{t}\right|e^{-\left|\partial_{x}I_{t}\right|}+\left|\partial_{y}d_{t}\right|e^{-\left|\partial_{y}I_{t}\right|}.$$

where $d_{t}$ denotes the disparity map corresponding to the predicted depth map $D_{t}$.

The depth and pose networks are jointly optimized using the photometric and smoothness objectives. The resulting depth-estimation loss is defined as follows:

(5) $$L_{\mathrm{DE}}=\lambda L_{p}+\mu L_{s},$$

where λ and μ control the contributions of photometric reconstruction and edge-aware smoothness, respectively. We set λ=1 and $\mu =10^{-3}$, retaining photometric consistency as the primary supervision while regularizing depth in weakly textured regions. For consistency, we adopt the multi-scale supervision scheme of Monodepth2 [15], with the scale weights following the original paper.

### 3.2. Nighttime Depth Estimation Network Architecture

**Figure 3:**
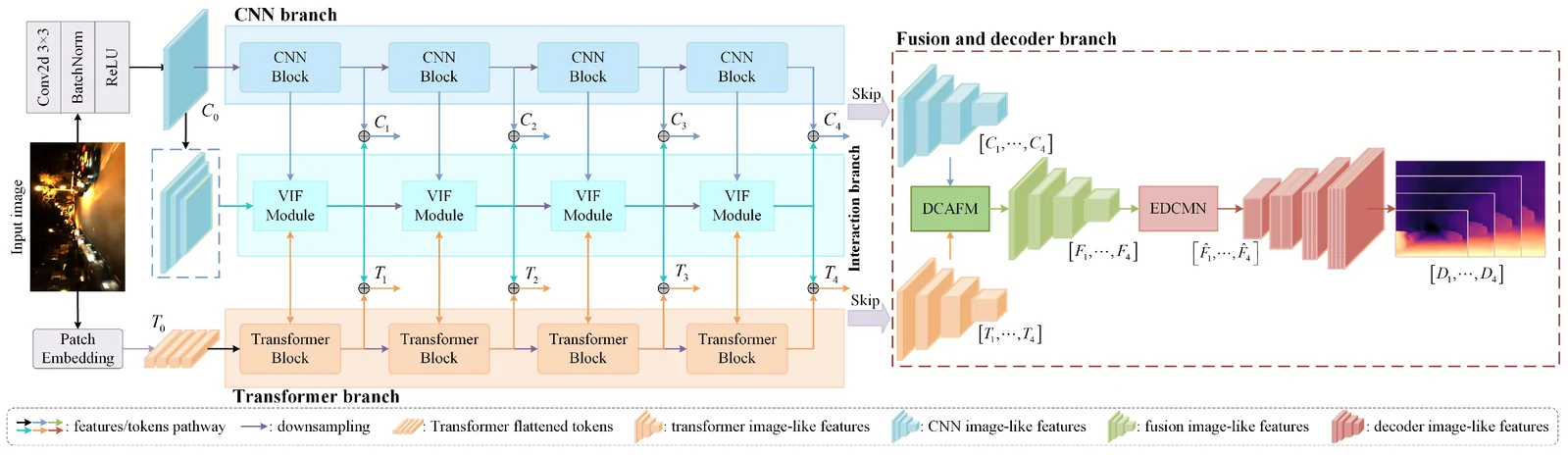
Overall architecture of DIDAF-Depth. The Transformer-CNN Vertical Interaction Fusion (TC-VIF) encoder couples a ResNet50 CNN branch [29] with ImageNet-pretrained weights and a Swin Transformer branch [30] through four Vertical Interaction Fusion (VIF) modules. The resulting feature sets are integrated by the Dual-Coupled Attentional Fusion Module (DCAFM) and decoded by the Edge-aware Densely Cascaded Multi-scale Network (EDCMN) into four depth predictions, ${\left\{D_{i}\right\}}_{i=1}^{4}$.

As shown in Figure 3, DIDAF-Depth contains three components: the TC-VIF encoder, DCAFM, and EDCMN. Together, these modules define the end-to-end inference procedure specified in Algorithm 1. At each of the four stages of the TC-VIF encoder, the CNN branch preserves spatially organized local structure, while the Transformer branch develops contextual representations from flattened tokens. Four Vertical Interaction Fusion (VIF) modules exchange information between the branches across the encoder stages to produce the paired feature pyramids ${\left\{C_{i}\right\}}_{i=1}^{4}$ and ${\left\{T_{i}\right\}}_{i=1}^{4}$. DCAFM integrates each feature pair into $F_{i}$, and it is applied at all four scales. EDCMN converts the fused pyramid into decoder features ${\left\{{\hat{F}}_{i}\right\}}_{i=1}^{4}$ and depth predictions ${\left\{D_{i}\right\}}_{i=1}^{4}$. The depth map $D_{4}$ is used as the final output for evaluation.
**Algorithm 1:** DIDAF-Depth inference workflow corresponding to Figure 3**Data:** target image $I_{t}$**Result:** final depth map $D_{4}$1   Generate initial branch representations $C_{0}$ by Conv–BN–ReLU and $T_{0}$ by patch embedding. 2   **for** i=1,…,4 **do** 3      Process $C_{i-1}$ and $T_{i-1}$ with the *i*-th TC-VIF stage. 4      Perform CNN-to-Transformer and Transformer-to-CNN interaction in VIF, and output $\left(C_{i},T_{i}\right)$. 5   **end for** 6   Apply DCAFM at each paired scale, $F_{i}=\mathrm{DCAFM}\left(C_{i},{\mathrm{map}}_{i}\left(T_{i}\right)\right)$, forming ${\left\{F_{i}\right\}}_{i=1}^{4}$. 7   Decode ${\left\{F_{i}\right\}}_{i=1}^{4}$ with EDCMN to obtain ${\left\{{\hat{F}}_{i}\right\}}_{i=1}^{4}$ and predict ${\left\{D_{i}\right\}}_{i=1}^{4}$ with depth heads. 8   Use $D_{4}$ as the final depth prediction for evaluation.

#### 3.2.1. Transformer-CNN Vertical Interaction Fusion Encoder

**Figure 4:**
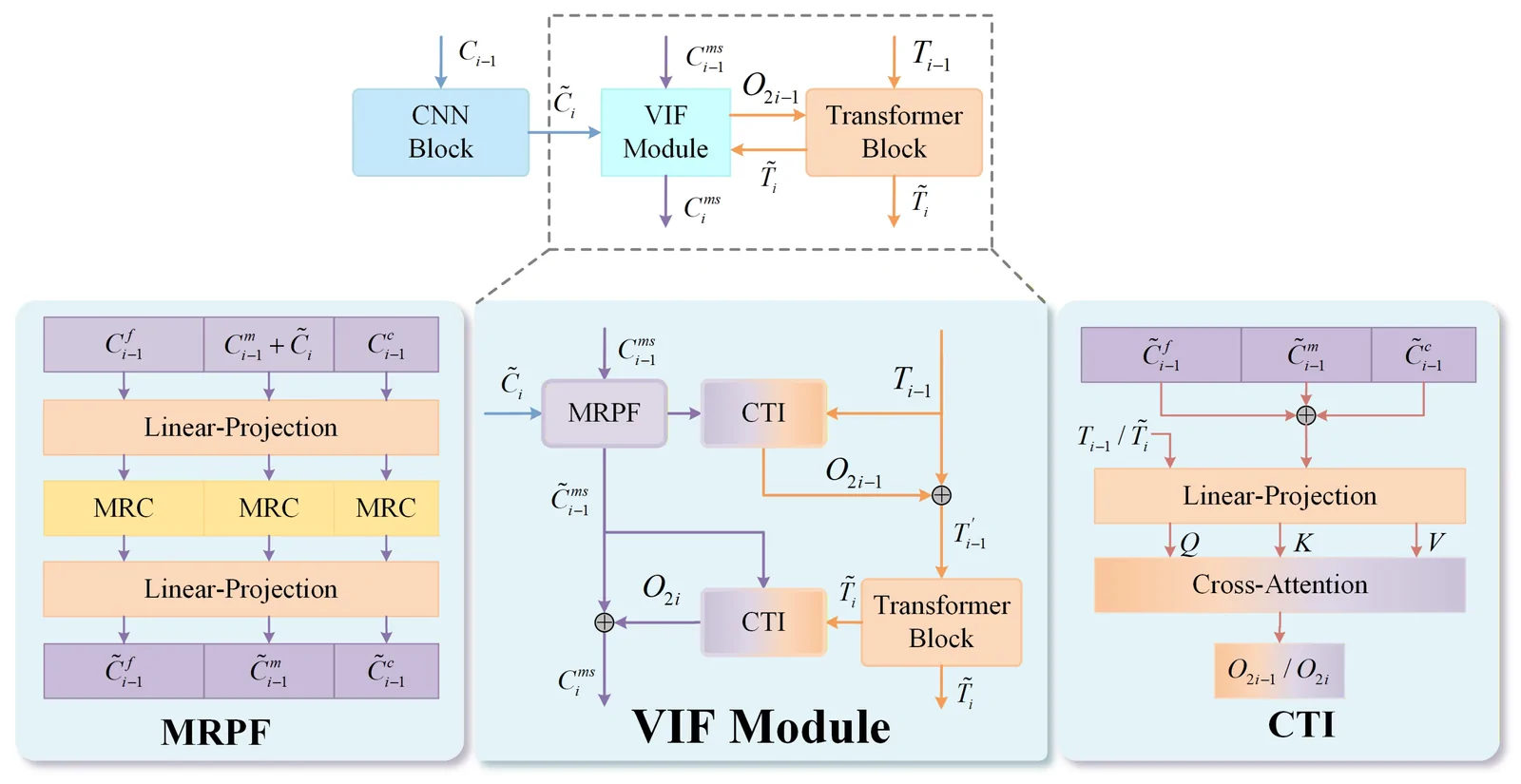
Single-stage organization of the Transformer-CNN Vertical Interaction Fusion (TC-VIF) encoder. The Vertical Interaction Fusion (VIF) module performs ordered CNN-to-Transformer and Transformer-to-CNN interaction through CNN–Transformer Interaction (CTI). Multi-Receptive-field Projection and Fusion (MRPF) prepares fine-, middle-, and coarse-scale CNN features with the same channel dimension, denoted by superscripts f, m, and c, respectively, where the fine and coarse features have twice and half the spatial resolution of the middle feature. MRC denotes Multi-Receptive-field Convolution using standard non-dilated 3 × 3 and 5 × 5 kernels.

The central operation at each stage of the TC-VIF encoder is a VIF module that performs ordered bidirectional interaction between the CNN and Transformer pathways, as illustrated in Figure 4. Let $C_{i-1}$ and $T_{i-1}$ denote the features entering the *i*-th stage. Here, *C*-features are image-shaped CNN feature maps, whereas *T*-features are Transformer tokens that retain a corresponding spatial grid. The CNN block first extracts a local convolutional representation:

(6) $${\tilde{C}}_{i}=B_{i}^{C}\left(C_{i-1}\right),$$

where $B_{i}^{C}\left(\cdot \right)$ denotes the *i*-th CNN block.

To provide scale-aware local evidence, VIF maintains a multi-scale CNN state $C_{i-1}^{\mathrm{ms}}=\left\{C_{i-1}^{f},C_{i-1}^{m},C_{i-1}^{c}\right\}$. The newly extracted feature ${\tilde{C}}_{i}$ has the same spatial and channel dimensions as the middle-scale feature and is therefore directly injected into this state. The resulting representation is refined by Multi-Receptive-field Projection and Fusion (MRPF):

(7) $$\begin{array}{c} {\tilde{C}}_{i-1}^{\mathrm{ms}}={\mathrm{MRPF}}_{i}\left(C_{i-1}^{f},\;C_{i-1}^{m}+{\tilde{C}}_{i},\;C_{i-1}^{c}\right), \end{array}$$

As detailed in the left panel of Figure 4, MRPF applies linear projection and multi-receptive-field convolution to enhance structural cues at different receptive fields. Before cross-branch interaction, the fine- and coarse-scale features are aligned to the middle scale and integrated with the middle-scale feature. This produces a compact CNN representation for interaction while preserving scale-complementary local information:

(8) $${\bar{C}}_{i}^{m}=P_{f}^{i}\left({\tilde{C}}_{i-1}^{f}\right)+{\tilde{C}}_{i-1}^{m}+P_{c}^{i}\left({\tilde{C}}_{i-1}^{c}\right),$$

where $P_{f}^{i}\left(\cdot \right)$ and $P_{c}^{i}\left(\cdot \right)$ denote the alignment functions that transform fine- and coarse-scale features to the middle-scale resolution and channel dimension.

The core operation in VIF is directed CNN–Transformer Interaction (CTI), shown in the right panel of Figure 4. For an interaction from a source feature *Y* to a target feature *X*, the target produces the query, whereas the source produces the key and value. This design updates the target representation using information selected from the source branch:

(9) $$\begin{array}{c} {\mathrm{CTI}}_{i}\left(X\leftarrow Y\right)=\psi_{i}\left(\mathrm{softmax}\left(\frac{\phi_{q}^{i}\left(X\right){\left[\phi_{k}^{i}\left(Y\right)\right]}^{⊤}}{\sqrt{d}}\right)\phi_{v}^{i}\left(Y\right)\right), \end{array}$$

where $\phi_{q}^{i}\left(\cdot \right)$, $\phi_{k}^{i}\left(\cdot \right)$, and $\phi_{v}^{i}\left(\cdot \right)$ denote the query, key, and value projections, respectively; $\psi_{i}\left(\cdot \right)$ denotes the output projection; and *d* denotes the channel dimension. ${\mathrm{tok}}_{i}\left(\cdot \right)$ denotes grid-preserving flattening of a CNN feature map into tokens for CTI, and ${\mathrm{map}}_{i}\left(\cdot \right)$ reshapes tokens back to the corresponding stage-*i* spatial grid when a feature map is required.

Using this formulation, each VIF module performs two ordered CTI operations. In the CNN-to-Transformer direction, $T_{i-1}$ is the target and ${\bar{C}}_{i}^{m}$ is the source. Local CNN evidence constrains the subsequent Transformer update and helps suppress attention responses caused by unreliable nighttime observations. The first interaction and the resulting Transformer update are formulated as follows:

(10) $$\left\{\begin{array}{cc} O_{2i-1} & ={\mathrm{CTI}}_{i}^{T\leftarrow C}(T_{i-1}\leftarrow {\mathrm{tok}}_{i}({\bar{C}}_{i}^{m})),\\ {\tilde{T}}_{i} & =B_{i}^{T}\left(T_{i-1}+\alpha_{i}O_{2i-1}\right), \end{array}\right.$$

where $O_{2i-1}$ denotes the CNN-guided Transformer interaction feature, $B_{i}^{T}\left(\cdot \right)$ denotes the *i*-th Transformer block, and $\alpha_{i}$ is a learnable scaling parameter controlling the interaction strength.

The updated Transformer representation then serves as the source for the reverse interaction, allowing global context to guide ambiguous local structures. The Transformer-to-CNN interaction and the resulting CNN update are as follows:

(11) $$\left\{\begin{array}{cc} O_{2i} & ={\mathrm{CTI}}_{i}^{C\leftarrow T}({\mathrm{tok}}_{i}({\bar{C}}_{i}^{m})\leftarrow {\tilde{T}}_{i}),\\ C_{i}^{\mathrm{ms}} & ={\tilde{C}}_{i-1}^{\mathrm{ms}}+R_{i}({\mathrm{map}}_{i}\left(O_{2i}\right)), \end{array}\right.$$

where $O_{2i}$ denotes the Transformer-guided CNN interaction feature, and $R_{i}\left(\cdot \right)$ maps the reshaped middle-scale interaction feature back to the multi-scale CNN state.

Finally, the stage-wise outputs are normalized and passed to the next encoding stage:

(12) $$\left\{\begin{array}{cc} C_{i} & =\mathrm{BN}({\tilde{C}}_{i}+C_{i}^{m}),\\ T_{i} & =\mathrm{LN}({\tilde{T}}_{i}+{\mathrm{tok}}_{i}(C_{i}^{m})), \end{array}\right.$$

where $C_{i}^{m}$ is the middle-scale component of the updated multi-scale CNN state $C_{i}^{\mathrm{ms}}$, and BN(·) and LN(·) denote batch normalization (BN) [31] and layer normalization (LN) [32], respectively. Thus, $C_{i}$ is an image-shaped feature map and $T_{i}$ remains token-shaped inside the Transformer branch. At stage $i,C_{i},C_{i}^{m},{\mathrm{map}}_{i}\left(T_{i}\right)\in R^{H_{i}\times W_{i}\times d_{i}}$ and $T_{i},{\mathrm{tok}}_{i}\left(C_{i}^{m}\right)\in R^{N_{i}\times d_{i}}$, where $N_{i}=H_{i}W_{i}$.

#### 3.2.2. Dual-Coupled Attentional Fusion Module

Although the TC-VIF encoder promotes interaction between the two branches, the Transformer and CNN features still encode complementary information in different forms, namely, global scene semantics and local texture and structure, which must be consolidated into a unified representation. Under nighttime conditions, sparse and unevenly distributed features make direct feature aggregation inadequate for reliably preserving these complementary cues. We therefore place a lateral fusion block, DCAFM, before the skip connections. Unlike prior methods [20,33] that perform fusion along either the channel or spatial dimension, and unlike single channel–spatial fusion routes such as GlocalFuse-Depth [19], DCAFM employs a dual-coupled pathway design that incorporates both dual spatial and dual channel attention pathways. This design jointly aligns Transformer and CNN representations across spatial and channel dimensions, enabling more effective feature utilization and collaborative modeling, and ultimately providing semantically consistent fused features for the EDCMN decoder.

**Figure 5:**
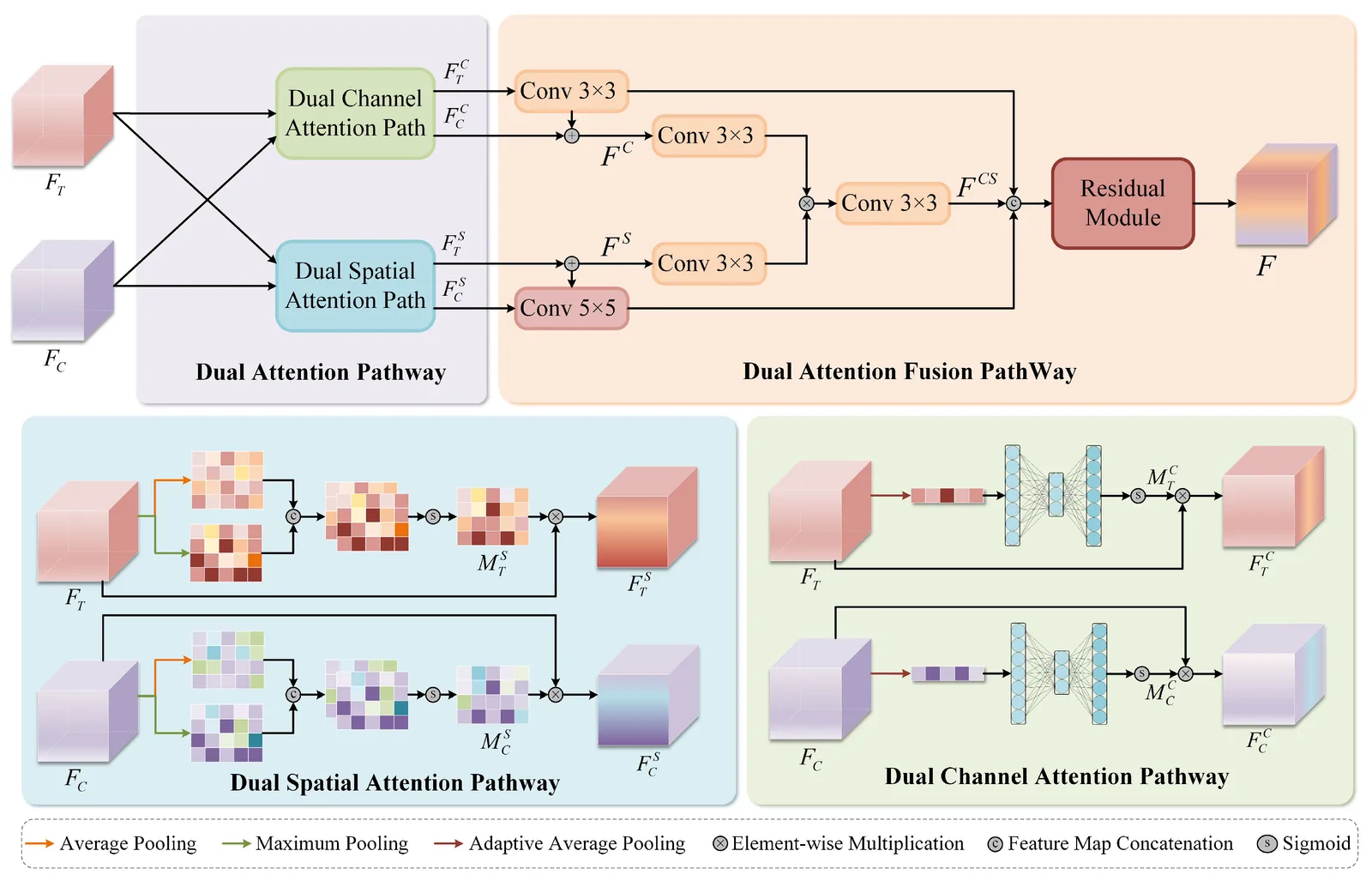
Structure of the Dual-Coupled Attentional Fusion Module (DCAFM). Subscripts C and T identify the CNN and Transformer branches, while superscripts C and S denote channel and spatial processing, respectively.

At the *i*-th stage, let $F_{C}=C_{i}$ and $F_{T}={\mathrm{map}}_{i}\left(T_{i}\right)$ denote the image-shaped CNN and Transformer features entering DCAFM, respectively, and DCAFM is applied independently at all four scales. For clarity, the stage index is omitted from the intermediate variables within DCAFM. As shown in Figure 5, we first extract global context by global average pooling (GAP) and produce channel weights using a multilayer perceptron (MLP) with two linear layers:

(13) $$\left\{\begin{array}{cc} M_{C}^{C} & =\sigma \left({\mathrm{MLP}}_{C}\left(\mathrm{GAP}(F_{C})\right)\right),\\ M_{T}^{C} & =\sigma \left({\mathrm{MLP}}_{T}\left(\mathrm{GAP}(F_{T})\right)\right), \end{array}\right.$$

where ${\mathrm{MLP}}_{C}$ and ${\mathrm{MLP}}_{T}$ contain an intermediate rectified linear unit (ReLU) activation, and σ(·) is the sigmoid function. The resulting weights re-emphasize informative channels:

(14) $$\left\{\begin{array}{cc} F_{C}^{C} & =F_{C}⊗M_{C}^{C},\\ F_{T}^{C} & =F_{T}⊗M_{T}^{C},\\ F^{C} & =F_{C}^{C}+{\mathrm{Conv}}_{3\times 3}\left(F_{T}^{C}\right), \end{array}\right.$$

where ⊗ denotes element-wise (Hadamard) multiplication, with broadcasting applied when the attention weights contain singleton spatial dimensions. The 3 × 3 convolution applied to $F_{T}^{C}$ enhances local characteristics that Transformers under-emphasize.

In parallel, we capture spatial saliency at complementary granularities using average- and max-pooled maps, followed by a large-kernel convolution to model wider spatial dependencies:

(15) $$\left\{\begin{array}{cc} M_{C}^{S} & =\sigma \left({\mathrm{Conv}}_{7\times 7}\left(\mathrm{Concat}\left[\mathrm{AvgPool}\left(F_{C}\right),\mathrm{MaxPool}\left(F_{C}\right)\right]\right)\right),\\ M_{T}^{S} & =\sigma \left({\mathrm{Conv}}_{7\times 7}\left(\mathrm{Concat}\left[\mathrm{AvgPool}\left(F_{T}\right),\mathrm{MaxPool}\left(F_{T}\right)\right]\right)\right), \end{array}\right.$$

where AvgPool(·) and MaxPool(·) denote average and maximum pooling, respectively. The spatial masks modulate features pixel-wise:

(16) $$\left\{\begin{array}{cc} F_{C}^{S} & =F_{C}⊗M_{C}^{S},\\ F_{T}^{S} & =F_{T}⊗M_{T}^{S},\\ F^{S} & =F_{T}^{S}+{\mathrm{Conv}}_{5\times 5}\left(F_{C}^{S}\right), \end{array}\right.$$

where the 5 × 5 convolution on $F_{C}^{S}$ expands the CNN receptive field to narrow the gap to Transformer spatial coverage.

After pathway-specific optimization, we refine $F^{C}$ and $F^{S}$ with 3 × 3 convolutions and couple them through element-wise multiplication to explicitly bind channel semantics and spatial saliency:

(17) $$F^{\mathrm{CS}}={\mathrm{Conv}}_{3\times 3}\left({\mathrm{Conv}}_{3\times 3}\left(F^{C}\right)⊗{\mathrm{Conv}}_{3\times 3}\left(F^{S}\right)\right).$$

Finally, we concatenate three features: $F^{\mathrm{CS}}$, the locally enhanced Transformer feature ${\mathrm{Conv}}_{3\times 3}\left(F_{T}^{C}\right)$, and the receptive-field–expanded CNN feature ${\mathrm{Conv}}_{5\times 5}\left(F_{C}^{S}\right)$. We then apply a residual block (ResBlk) to produce the fused feature *F*:

(18) $$F=\mathrm{ResBlk}\left(\mathrm{Concat}\left[{\mathrm{Conv}}_{3\times 3}\left(F_{T}^{C}\right),F^{\mathrm{CS}},{\mathrm{Conv}}_{5\times 5}\left(F_{C}^{S}\right)\right]\right),$$

where ResBlk consists of two 3 × 3 convolutions with ReLU and an identity skip, and the output *F* corresponds to $F_{i}$ at the current scale.

#### 3.2.3. Edge-Aware Densely Cascaded Multi-Scale Network

**Figure 6:**
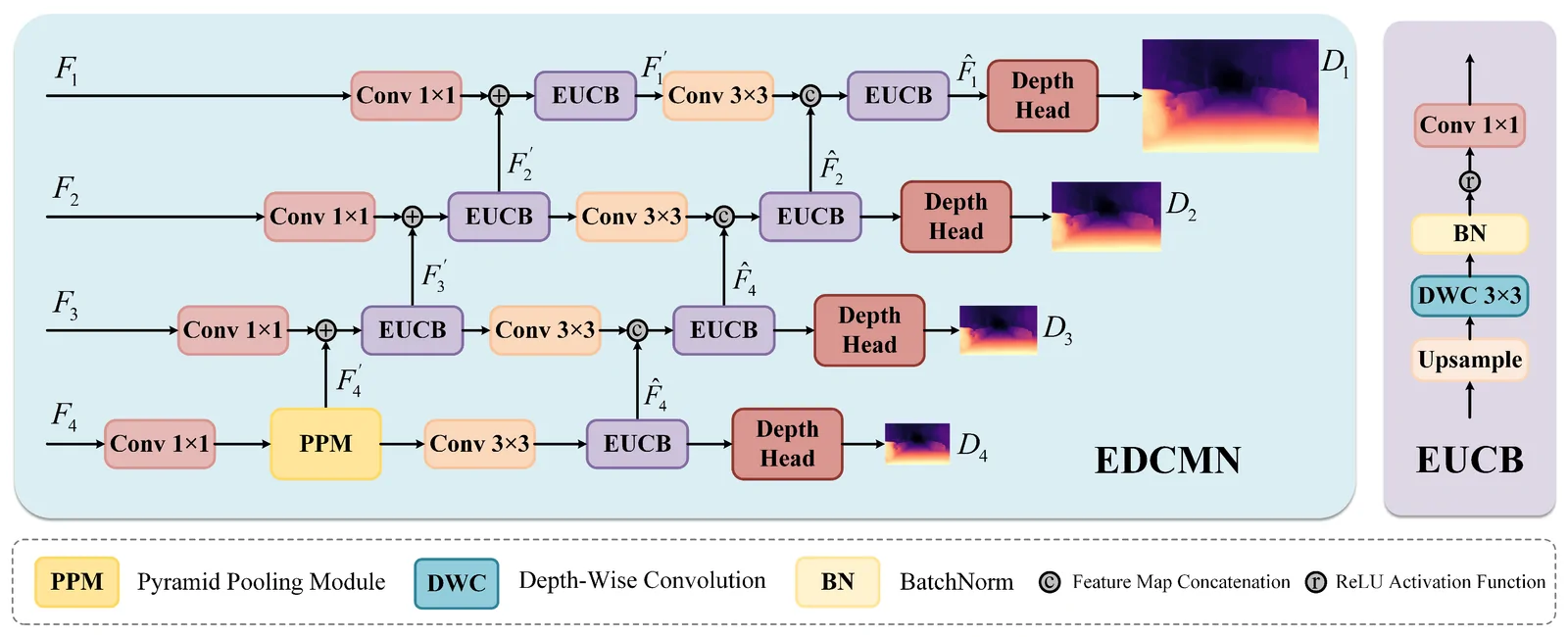
Structure of the Edge-aware Densely Cascaded Multi-scale Network (EDCMN). EUCB denotes the Edge-aware Upsampling Context Block.

Nighttime depth decoding must recover spatial resolution without losing scene-level depth organization or weak structural boundaries. Under low contrast, glare, and sparse texture, conventional U-Net-style decoding with sparse skip connections can progressively attenuate semantic guidance and local structure during upsampling. We therefore construct EDCMN as a coordinated coarse-to-fine reconstruction pathway, as shown in Figure 6. Rather than reconstructing each scale primarily from its local skip feature, EDCMN anchors decoding to a scene-level prior extracted at the coarsest resolution by the Pyramid Pooling Module (PPM) [34] and propagates this prior through the Feature Pyramid Network (FPN) [35]. As the representation moves toward finer scales, dense connections (DC) [36] carry forward the structural evidence accumulated along the decoding path, so that each Edge-aware Upsampling Context Block (EUCB) [37] refines boundaries within an increasingly informed representation. In this way, resolution recovery is guided by progressively enriched context, reducing the reliance on weak nighttime appearance cues at any individual scale.

Let ${\left\{F_{i}\right\}}_{i=1}^{4}$ denote the DCAFM outputs from fine to coarse scales, where i=1 corresponds to 1/2 resolution and i=4 corresponds to 1/16 resolution. As shown in Figure 6, EDCMN decodes this fused pyramid from coarse to fine. We use $F_{i}^{\prime }$ to denote the feature propagated along the FPN pathway and ${\hat{F}}_{i}$ to denote the densely refined decoder feature used for depth prediction. At the coarsest scale, $F_{4}$ is first compressed and enriched by PPM with multi-scale pooling bins, specifically 1 × 1, 2 × 2, 3 × 3, and 6 × 6:

(19) $$F_{4}^{\prime }=\mathrm{PPM}\left({\mathrm{Conv}}_{1\times 1}\left(F_{4}\right)\right).$$

This operation introduces global priors that remain useful when local nighttime cues are weak or unreliable. The resulting FPN feature is then refined by a 3 × 3 convolution and upsampled through EUCB to obtain the initial densely refined decoder feature:

(20) $${\hat{F}}_{4}=\mathrm{EUp}\left({\mathrm{Conv}}_{3\times 3}\left(F_{4}^{\prime }\right)\right),$$

where EUp(·) denotes the upsampling enhancement operation of EUCB. As illustrated on the right side of Figure 6, EUCB consists of upsampling, depth-wise convolution, batch normalization, ReLU activation, and a 1 × 1 convolution. This block improves spatial resolution while reinforcing edge-sensitive local structure.

At each shallower scale, FPN fuses the lateral DCAFM feature with the feature propagated from the deeper FPN stage. The summed representation is then processed by EUCB, allowing coarse semantic guidance to inform local reconstruction in dark or texture-sparse regions:

(21) $$F_{i}^{\prime }=\mathrm{EUp}\left({\mathrm{Conv}}_{1\times 1}\left(F_{i}\right)+F_{i+1}^{\prime }\right),$$

where i∈{1,2,3}.

EDCMN further uses dense cascading to preserve structural information across decoding stages. At each scale, the locally refined FPN feature is concatenated with the densely refined feature from the deeper stage and processed by another EUCB. This dense link reduces information decay and repeatedly strengthens boundary and structure recovery during upsampling:

(22) $${\hat{F}}_{i}=\mathrm{EUp}\left(\mathrm{Concat}\left[{\mathrm{Conv}}_{3\times 3}\left(F_{i}^{\prime }\right),{\hat{F}}_{i+1}\right]\right),$$

where i∈{1,2,3}.

Finally, the densely refined feature at each scale is mapped to a depth prediction by the corresponding depth head $H_{i}$:

(23) $$D_{i}=H_{i}\left({\hat{F}}_{i}\right),$$

where i∈{1,2,3,4}; $H_{i}$ consists of a 3×3 convolution followed by a sigmoid activation; and $D_{i}$ denotes the predicted depth map at the *i*-th scale.

## 4. Results and Discussion

### 4.1. Datasets

**RobotCar-Night.** The Oxford RobotCar dataset [38], released in 2017, contains over 100 repeated traversals of fixed routes in Oxford, UK, collected between 2014 and 2015. Following [21,24,39] and using the training/testing split of [25], we build the RobotCar-Night subset from front stereo camera images of sequence 2014-12-16-18-44-24. For self-supervised training, only temporally consecutive monocular frames from this sequence are used, and stereo image pairs are not used for photometric supervision. The training set consists of 19k images from the first five segments (stationary frames removed), and the test set contains 411 images from the remaining segment. Ground-truth depth maps are produced using the official toolbox by fusing front LMS laser and INS data. Images are center-cropped from 1280 × 960 to 1152 × 640 to remove the car hood, and they are then resized to 576 × 320 for network input; the camera intrinsic matrix *K* is adjusted according to the crop offset and the horizontal and vertical resize factors.

**nuScenes-Night.** The nuScenes dataset [40], developed by Motional, comprises 1000 driving scenes captured in Boston and Singapore. Each scene lasts 20 s, yielding approximately 1.4 million images. Following the training/testing split of [25] and the setting of [41], we construct the nuScenes-Night subset using front camera images from 44 nighttime scenes. The same temporal monocular-frame protocol is adopted for self-supervised training: the training set comprises about 10k images from these scenes, while the test set contains 500 images from other nighttime scenes. Ground-truth depth maps are generated via the official toolbox using front LiDAR data. The original 1600 × 900 images are resized to 768 × 384 for network input, and the corresponding camera intrinsics are scaled to the network-input resolution.

### 4.2. Implementation Details

**Auxiliary Training.** Following [25], we use the daytime adversarial auxiliary strategy introduced by RNW [24] only during the first 20 epochs. This stage stabilizes the initial depth–pose optimization when low illumination, glare, and texture loss make temporal correspondences unreliable; the remaining training follows the monocular temporal-frame objective described in Section 3.1.

**Hyperparameters.** Our model is implemented in PyTorch version 2.0.0 [42] and trained for 40 epochs on two NVIDIA RTX 4090 GPUs. We employ the Adam optimizer [43] ($\beta_{1}=0.9$, $\beta_{2}=0.999$) with batch size 4. The initial learning rate is $10^{-4}$ and is halved at the 15th epoch.

**Evaluation Metrics.** Following [24,25,39,41], we adopt seven standard metrics: Abs Rel, Sq Rel, RMSE, ${\mathrm{RMSE}}_{\log}$, $\delta_{1}$, $\delta_{2}$, and $\delta_{3}$. Following the STEPS evaluation protocol [25], predictions are resized to the cropped ground-truth resolution and valid pixels are selected within the relevant depth cap. Median scaling is then applied, and the scaled predictions are clipped before metric computation. The metrics are defined as follows:

(24) $$\left\{\begin{array}{c} \mathrm{Abs}\;\mathrm{Rel}=\frac{1}{\left|\Omega \right|}\sum_{p\in \Omega }\frac{\left|{\hat{D}}_{p}-D_{p}^{*}\right|}{D_{p}^{*}},\\ \mathrm{Sq}\;\mathrm{Rel}=\frac{1}{\left|\Omega \right|}\sum_{p\in \Omega }\frac{{\left({\hat{D}}_{p}-D_{p}^{*}\right)}^{2}}{D_{p}^{*}},\\ \mathrm{RMSE}=\sqrt{\frac{1}{\left|\Omega \right|}\sum_{p\in \Omega }{\left({\hat{D}}_{p}-D_{p}^{*}\right)}^{2}},\\ {\mathrm{RMSE}}_{\log}=\sqrt{\frac{1}{\left|\Omega \right|}\sum_{p\in \Omega }{\left(\log{\hat{D}}_{p}-\log D_{p}^{*}\right)}^{2}},\\ \delta_{i}=\frac{1}{\left|\Omega \right|}\left|\left\{p\in \Omega \;|\;\max\left(\frac{{\hat{D}}_{p}}{D_{p}^{*}},\frac{D_{p}^{*}}{{\hat{D}}_{p}}\right)<1.25^{i}\right\}\right|,\;i\in \left\{1,2,3\right\}. \end{array}\right.$$

Here, ${\hat{D}}_{p}$ and $D_{p}^{*}$ denote the predicted and ground-truth depth values at valid pixel p, respectively; Ω is the set of valid pixels in an image; and *i* indexes the three threshold accuracies.

### 4.3. Quantitative Results

**RobotCar-Night Results.** Table 1 presents the performance of DIDAF-Depth and existing methods on the RobotCar-Night dataset. The results show that daytime methods, including Monodepth2 [15], SwinDepth [16], and Lite-Mono [44], suffer from severe performance degradation under nighttime conditions, whereas specialized nighttime approaches achieve clear improvements. Compared with STEPS [25], our method consistently improves both evaluation ranges, reducing Abs Rel error and increasing $\delta_{1}$ accuracy by a clear margin. These gains demonstrate the effectiveness of the proposed dual-path interaction and dual-attention fusion architecture in handling sparse nighttime visual information.

**Figure 7:**
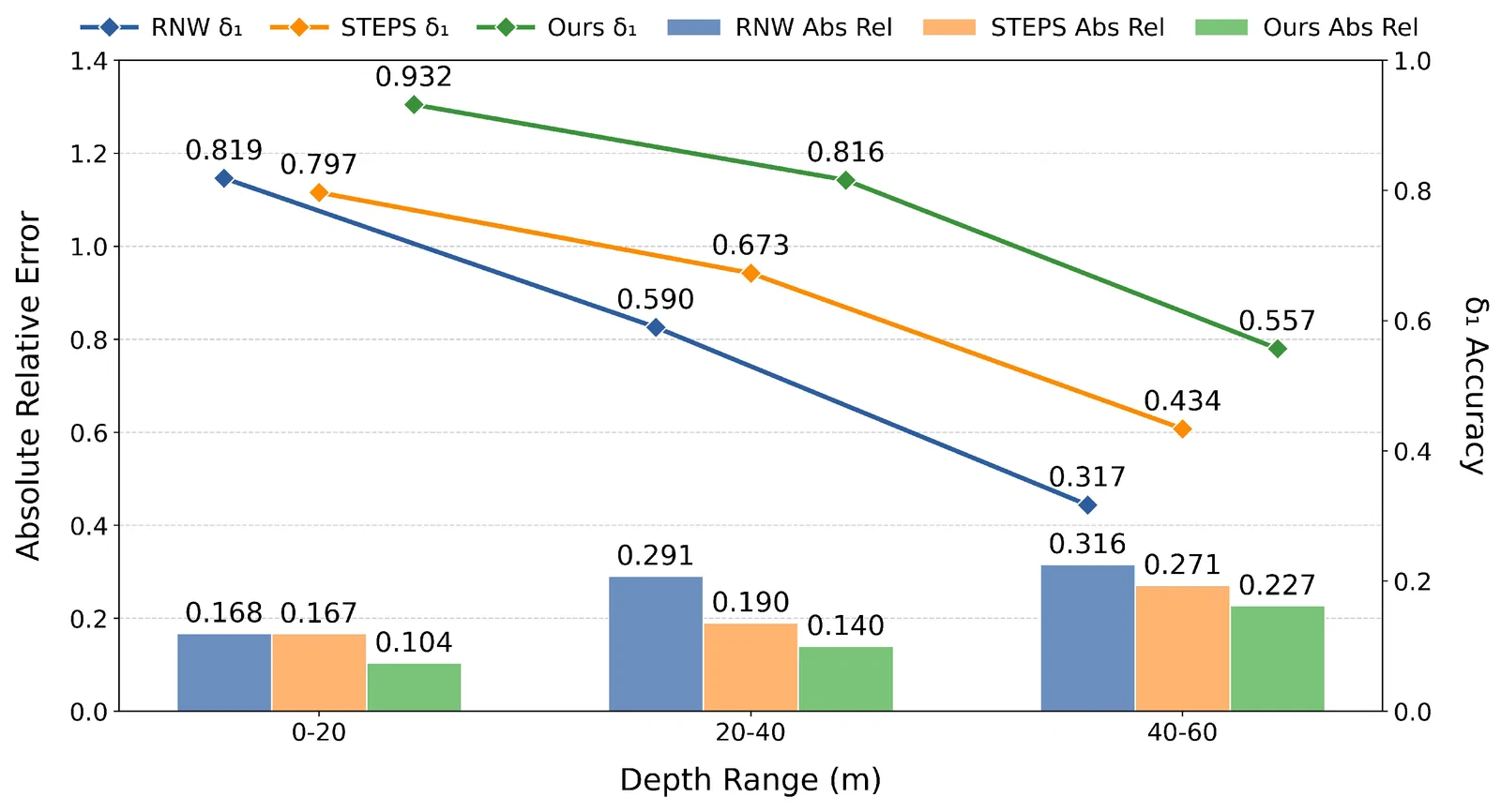
Range-wise comparison of RNW [24], STEPS [25], and DIDAF-Depth on RobotCar-Night over 0–20, 20–40, and 40–60 m intervals. Bars report Abs Rel (left axis, lower is better), and lines report threshold accuracy $\delta_{1}$ (right axis, higher is better).

Figure 7 presents the range-wise evaluation on RobotCar-Night by dividing the evaluated depth into near-, middle-, and far-range intervals. As the evaluated depth range increases, all methods exhibit a noticeable performance degradation, reflecting the increasing difficulty of nighttime depth estimation under weakened illumination, sparse textures, and ambiguous object boundaries. Nevertheless, DIDAF-Depth maintains lower Abs Rel and higher $\delta_{1}$ than the competing methods in every depth interval. This indicates that the proposed method can not only exploit relatively reliable visual cues in nearby regions but also maintain more stable depth estimation when geometric evidence becomes sparse and uncertain at longer distances.

**nuScenes-Night Results.** Table 2 evaluates DIDAF-Depth under the more diverse urban layouts and lighting conditions of nuScenes-Night. Compared with RobotCar-Night, this setting introduces stronger scene variation, denser traffic, and more frequent headlight interference. DIDAF-Depth achieves the best results at both 40 m and 60 m. Relative to STEPS [25], it reduces RMSE by 13.4% at 40 m and 12.5% at 60 m, while also improving $\delta_{1}$ accuracy at both depth caps. These results show that the proposed architecture remains effective when nighttime degradation is coupled with more complex traffic scenes.

We further assess cross-domain transfer by training on RobotCar-Night and testing directly on nuScenes-Night, as reported in Table 3. This setting evaluates robustness to changes in city layout, camera configuration, scene density, and illumination distribution. Compared with STEPS, DIDAF-Depth reduces Abs Rel by 28.8% and RMSE by 21.7%, while improving $\delta_{1}$ accuracy by 21.8%. It also improves $\delta_{1}$ over SRNSD [41] by 29.1%. These gains indicate that the learned local-global interaction and edge-aware decoding are not limited to the source nighttime domain.

Finally, we examined the computational profile of DIDAF-Depth on RobotCar-Night to place the accuracy gains in a practical context. At the experimental input resolution of 320 × 576 with batch size 1, the depth-inference path has 56.954 M parameters and 422.170 G FLOPs. On a single NVIDIA GeForce RTX 5090 GPU, it processes one image in 68.259 ms, corresponding to 14.65 FPS, with 1713.4 MB peak allocated CUDA memory. The main cost comes from maintaining complementary CNN and Transformer representations, whose branch encoders together account for 47.337 M parameters. In contrast, the proposed VIF, DCAFM, and EDCMN modules add 1.814 M, 6.686 M, and 1.118 M parameters, respectively. These measurements indicate that DIDAF-Depth trades lightweight real-time efficiency for more reliable nighttime depth recovery, while the added interaction, fusion, and structure-aware decoding modules remain modest relative to the dual-branch backbone.

### 4.4. Qualitative Results

**Figure 8:**
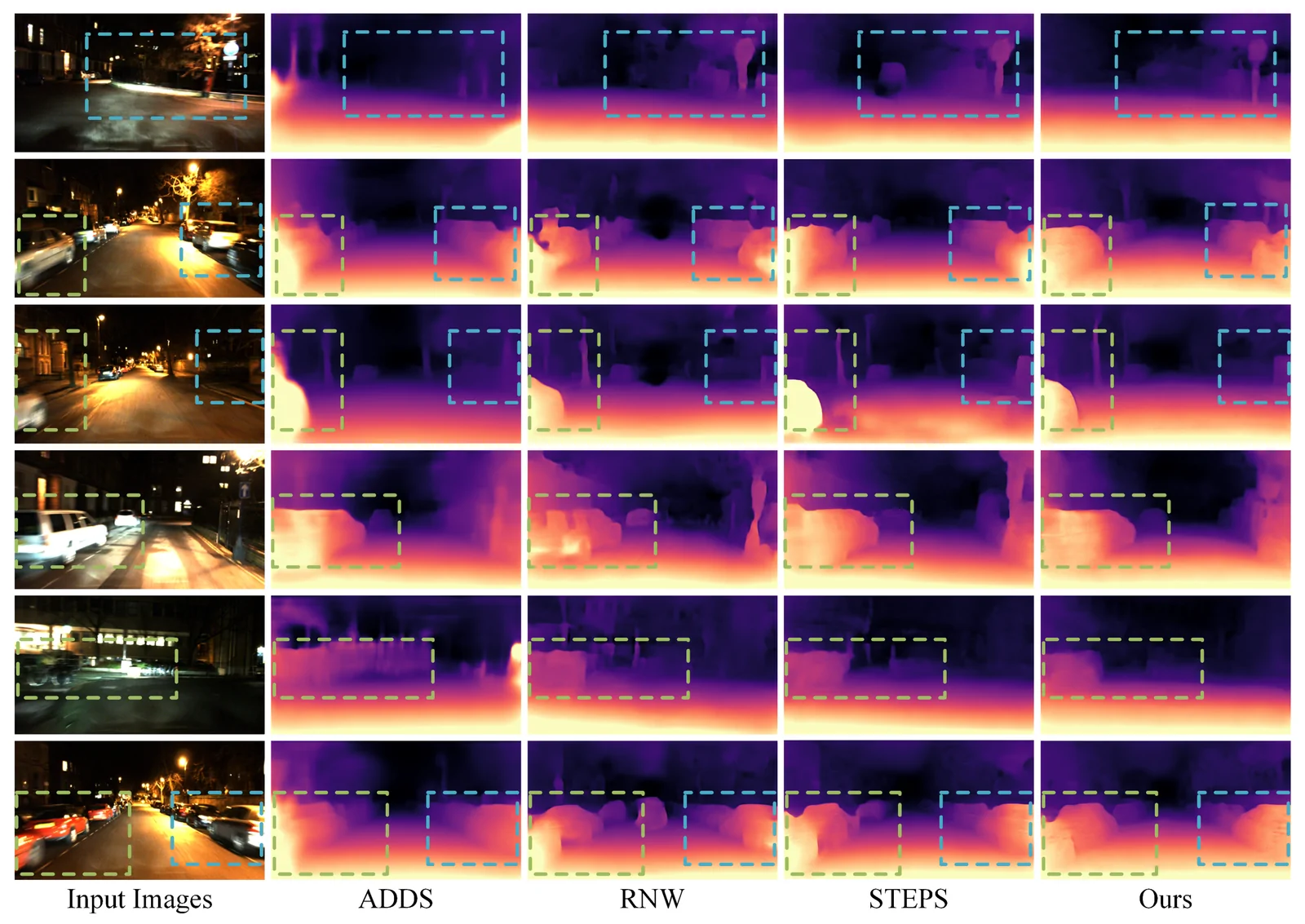
Qualitative depth comparison on RobotCar-Night. Columns from left to right show input images and predictions from ADDS [39], RNW [24], STEPS [25], and DIDAF-Depth. Colored dashed boxes mark selected object boundaries, thin structures, and illumination-affected regions.

**Figure 9:**
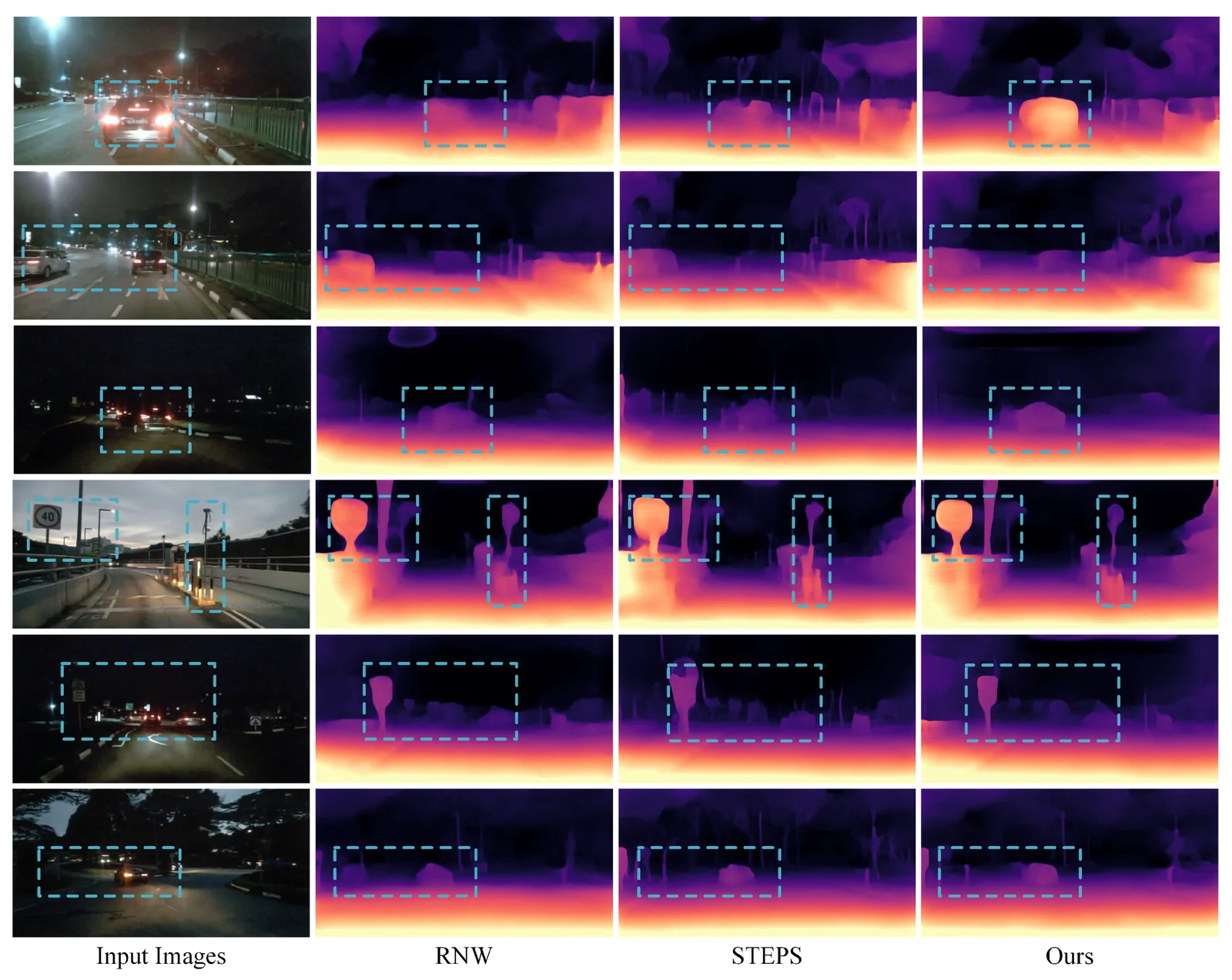
Qualitative depth comparison on nuScenes-Night. Columns from left to right show input images and predictions from RNW [24], STEPS [25], and DIDAF-Depth. Cyan dashed boxes mark vehicles, roadside structures, and low-visibility regions.

Figure 8 and Figure 9 compare depth predictions under complementary nighttime conditions. On RobotCar-Night (Figure 8), the principal differences appear around vehicles, roadside structures, and transitions between the illuminated road and dark background. ADDS [39] tends to smooth these transitions into broad depth regions, while RNW [24] introduces irregular responses that disturb scene layering. STEPS [25] recovers a more coherent layout but still weakens some contours in dark or glare-affected areas. DIDAF-Depth preserves clearer vehicle boundaries and thin roadside structures while maintaining a smoother progression from nearby roads to distant regions, as highlighted by the dashed boxes.

Figure 9 examines the same properties in scenes with dimmer backgrounds, stronger headlights, and more varied road geometry. Under these conditions, competing predictions often merge vehicles with their surroundings or produce unstable shapes near light sources, making foreground–background separation ambiguous. DIDAF-Depth maintains more complete vehicle silhouettes and roadside objects across both illuminated and poorly visible regions. These consistent qualitative gains across both datasets suggest that the proposed design enhances the structural reliability of nighttime depth estimation under diverse illumination degradations and scene complexities.

### 4.5. Ablation Study

We organize the ablation study at two levels to identify where the performance gains originate. The first analysis evaluates the complementarity of the TC-VIF encoder, DCAFM, and EDCMN within the complete architecture. We then isolate the component choices within the encoder, fusion module, and decoder. All experiments are conducted on RobotCar-Night with a maximum depth of 60 m. Here, “TC” denotes a parallel Transformer-CNN encoder without VIF.

**Core-component complementarity.** Table 4 first establishes how the three components contribute to the complete architecture. The added parameter column shows that the complete model is not the largest configuration, because EDCMN replaces the standard decoder with a lighter structure-aware decoder. At matched DCAFM fusion, replacing the TC encoder with the TC-VIF encoder reduces Abs Rel by 5.6% and improves $\delta_{1}$ by 2.8%, isolating the benefit of vertical interaction. When DCAFM is omitted, adding EDCMN to the TC-VIF encoder reduces Abs Rel by 4.1% relative to the encoder-only baseline. Using DCAFM without EDCMN yields an 8.2% reduction. Together, these comparisons indicate that decoding and fusion improve different parts of the pipeline. EDCMN then reduces Abs Rel by another 13.4% and improves $\delta_{1}$ by 4.0%, indicating that stronger fused representations still require structure-aware decoding.

**Figure 10:**
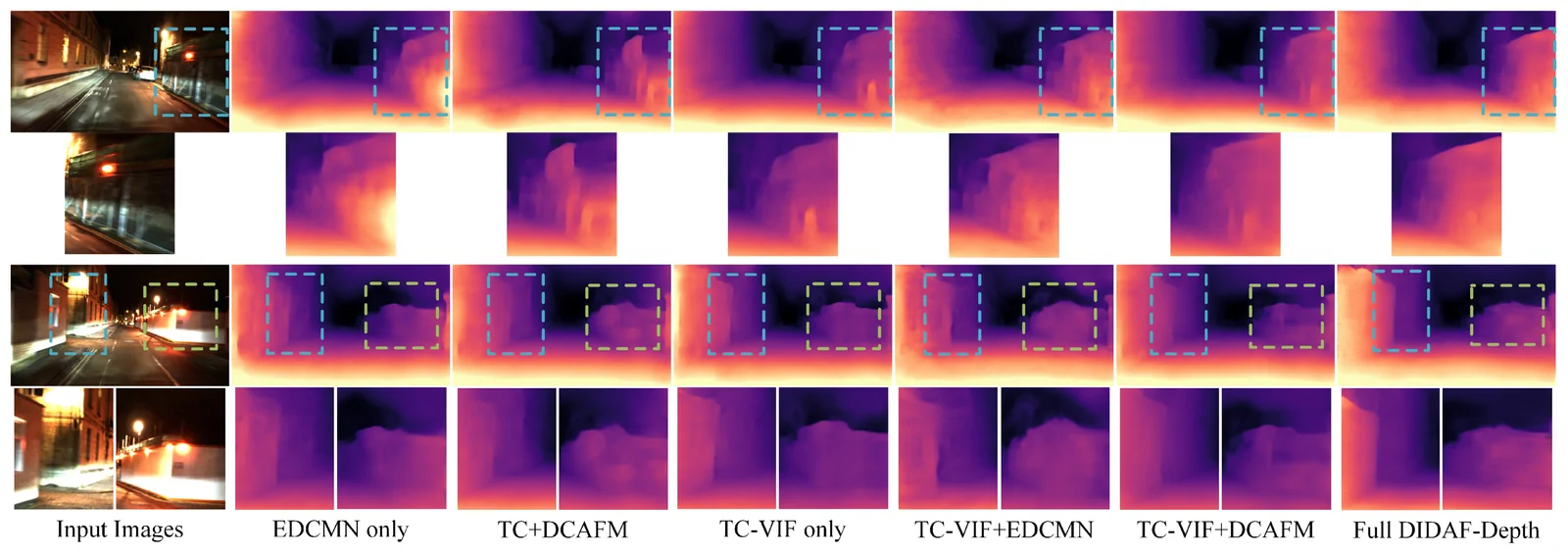
Qualitative component ablation on RobotCar-Night. Columns compare input images, EDCMN-only decoding, the parallel Transformer-CNN encoder combined with DCAFM, TC-VIF alone, TC-VIF combined with EDCMN, TC-VIF combined with DCAFM, and the full DIDAF-Depth model. TC denotes the parallel Transformer-CNN encoder without VIF. Dashed boxes and enlarged crops highlight boundary- and structure-sensitive regions affected by glare, weak texture, and low illumination.

Figure 10 complements this quantitative progression by revealing how the errors change across configurations. Encoder interaction and attentive fusion progressively improve scene-level depth organization, whereas the complete decoder most clearly restores vehicle contours and thin structures in poorly illuminated regions. The agreement between the two forms of evidence motivates the following module-level analyses.

**Vertical interaction in the encoder.** Having established its contribution at the system level, we next isolate the encoder architecture. As shown in Table 5, the TC-only configuration performs worse than both single-branch baselines, indicating that without interaction the two branches can interfere with each other and further degrade feature quality. By introducing interaction, the TC-VIF encoder reduces Abs Rel by 17.0%, 9.9%, and 36.5% relative to the CNN-only, Transformer-only, and TC baselines, respectively. It also exceeds the three representative Transformer-CNN architectures in Figure 2, with $\delta_{1}$ gains ranging from 2.8% to 17.0%. Thus, the improvement cannot be attributed merely to using two branches; it depends on the ordered bidirectional exchange between local and global representations.

**Figure 11:**
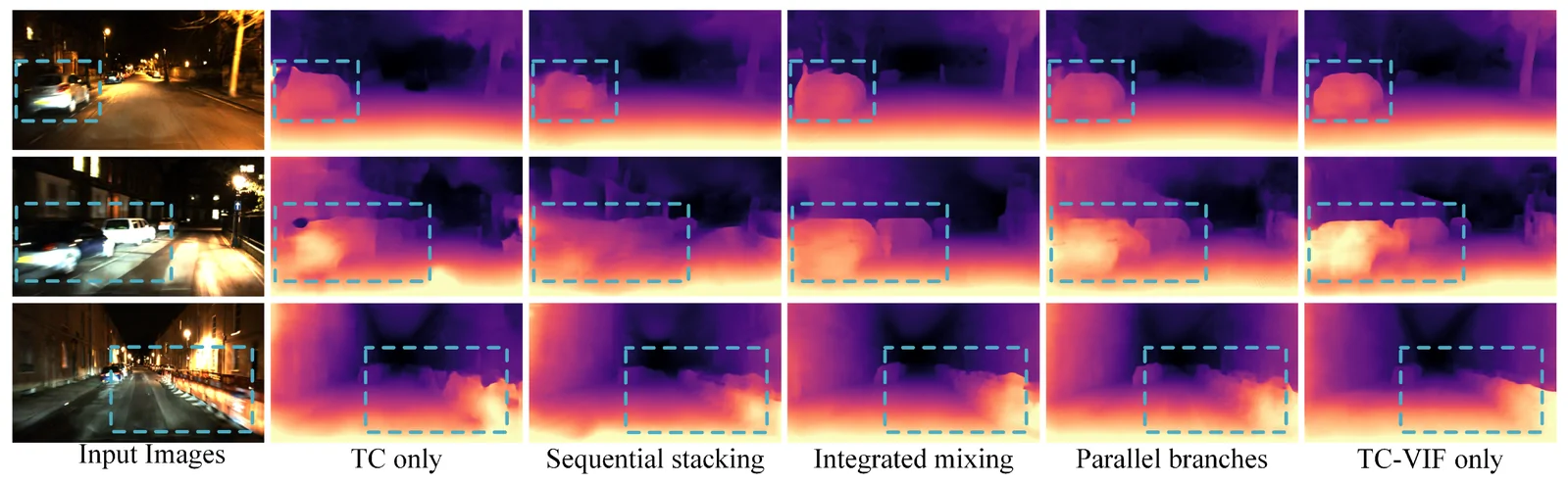
Qualitative comparison of Transformer-CNN encoder variants on RobotCar-Night. The columns compare representative hybrid encoder designs and the proposed TC-VIF encoder under the same nighttime depth-estimation setting. Cyan dashed boxes mark vehicles, roadside structures, and low-visibility regions.

Figure 11 shows the corresponding qualitative effect. Architectures without repeated bidirectional interaction exhibit fragmented vehicle contours or incomplete distant structures, particularly where nighttime texture is weak. The TC-VIF encoder preserves scene organization and sharper local boundaries through coordinated Transformer context and CNN detail.

**Dual-coupled attentional fusion.** With the encoder fixed, Table 6 examines how heterogeneous features should be fused. Relative to complete DCAFM, restricting fusion to channel or spatial attention increases Abs Rel by 30.2% and 25.9%, respectively, while assigning a single attention type to each branch remains inferior to joint channel-spatial processing. Removing residual refinement produces a comparable degradation, and replacing the multiplicative fusion in Equation (17) with simple feature-map addition improves Sq Rel but remains weaker in overall accuracy than the complete module. Relative to GlocalFuse-Depth fusion [19], DCAFM reduces Abs Rel by 10.8% and improves $\delta_{1}$ by 3.1%. Together, these comparisons show that the gain arises from coupling complementary attention pathways before cross-dimensional integration, rather than from attention or feature aggregation alone.

**Figure 12:**
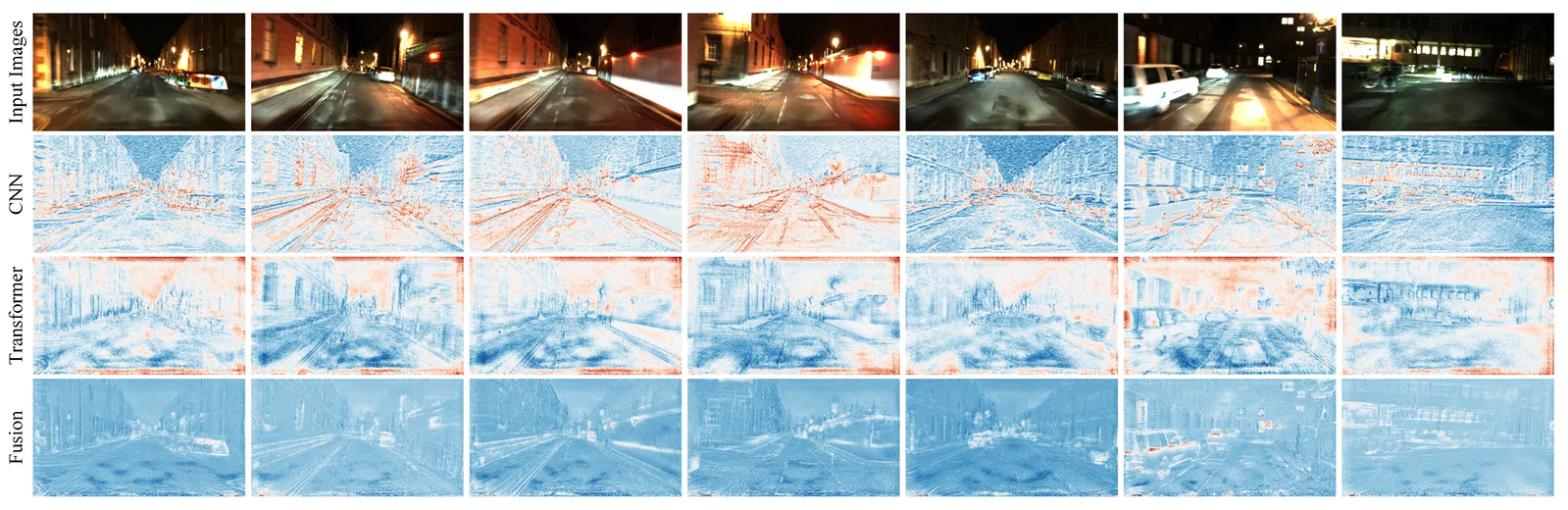
Feature-response visualization before and after DCAFM fusion on RobotCar-Night. Rows show input images, CNN features, Transformer features, and fused features after DCAFM.

Figure 12 provides complementary evidence at the representation level. CNN responses emphasize local texture, whereas Transformer responses cover broader structures but retain spurious edge activations. The fused responses preserve the wider spatial organization while concentrating activation around informative details and suppressing false boundaries. This pattern is consistent with the quantitative advantage of collaborative channel-spatial modeling.

**Figure 13:**
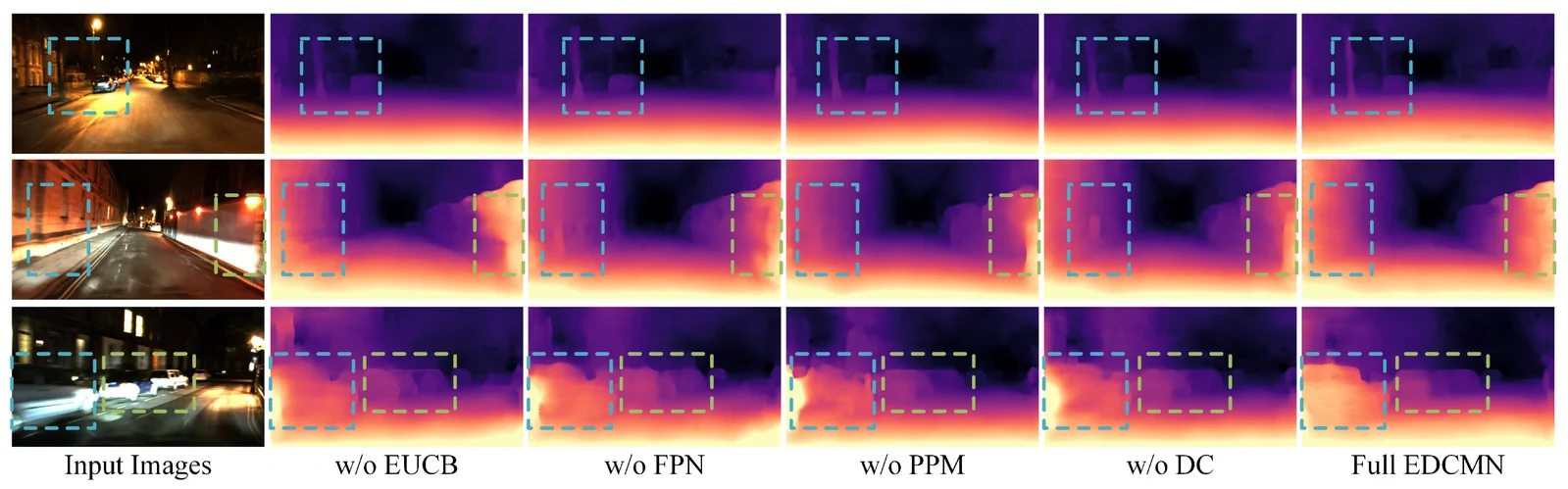
Qualitative decoder ablation on RobotCar-Night. Columns compare depth predictions after removing individual EDCMN components, including EUCB, FPN, PPM, and dense connections (DC). Colored dashed boxes highlight object boundaries and regions affected by glare or sparse texture.

**Edge-aware multi-scale decoding.** Finally, we fix the encoder and fusion stages and remove each EDCMN component individually. As shown in Table 7 and Figure 13, removing FPN or PPM increases Abs Rel by 29.31% and 25.00%, respectively, and introduces broad depth deviations across roads, vehicles, and foreground regions. These changes reflect the importance of cross-scale fusion and coarse contextual guidance when nighttime appearance cues are unreliable. Removing EUCB or DC increases Abs Rel by 14.66% and 17.24%, with more localized boundary blur and structural discontinuities. The two groups therefore play complementary roles: FPN and PPM stabilize scene-level depth organization, whereas EUCB and dense propagation preserve edges and structural continuity during upsampling.

**Figure 14:**
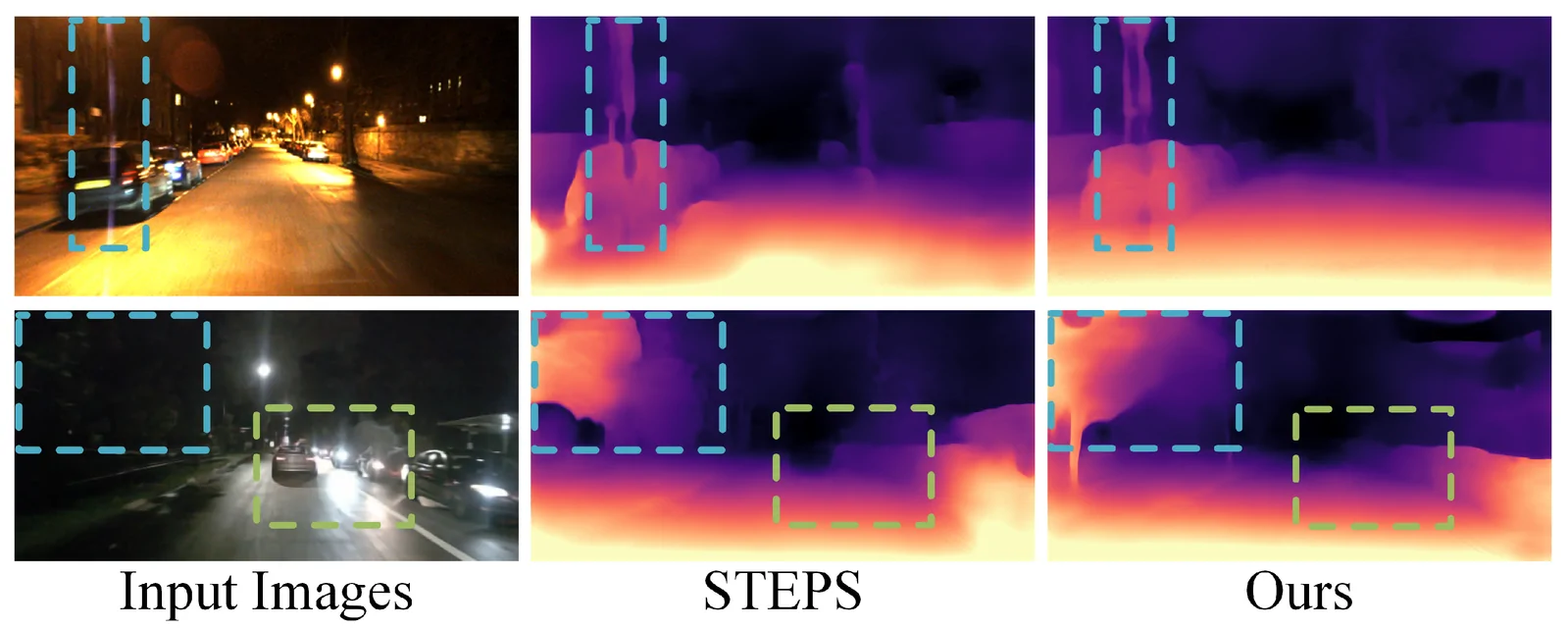
Typical failure cases of STEPS and DIDAF-Depth on RobotCar-Night under extreme darkness and severe overexposure or glare. Columns show input images, STEPS predictions, and DIDAF-Depth predictions. Dashed boxes mark regions where both methods can produce unreliable depth structures because visual cues are largely missing or saturated.

**Limitations.** As shown in Figure 14, both STEPS and DIDAF-Depth can still fail when images contain large extremely dark regions or severe local overexposure/glare. In these cases, the available photometric cues become too weak or distorted to support stable correspondence. Future work could improve robustness by introducing stronger exposure-aware augmentation, uncertainty-aware supervision, and more explicit handling of saturated or textureless regions.

## 5. Conclusions

This study investigates nighttime self-supervised monocular depth estimation from the perspective of architectural feature collaboration. Instead of mainly relying on image enhancement or domain transfer, DIDAF-Depth reorganizes the encoder–fusion–decoder pipeline around the complementary roles of convolutional locality and Transformer context. Specifically, the TC-VIF encoder promotes progressive cross-layer calibration between local and global representations, DCAFM alleviates residual spatial–channel mismatch during lateral fusion, and EDCMN preserves multi-scale structural cues through dense propagation and edge-aware upsampling. These components jointly form a unified architecture for sparse, glare-affected, and low-contrast nighttime scenes. Experiments on RobotCar-Night and nuScenes-Night demonstrate that explicit local–global interaction, attentive feature fusion, and edge-aware decoding are effective for nighttime depth estimation. Future work will explore how the proposed interaction and decoding principles can be extended to broader adverse conditions, such as rain, fog, mixed illumination, and more diverse deployment environments.

## Figures and Tables

**Table 1:** Quantitative comparison on RobotCar-Night at maximum depths of 40 and 60 m. Arrows indicate the preferred metric direction. ↓ denotes that lower values are better, whereas ↑ denotes that higher values are better. Bold marks the best result at each depth cap. An asterisk identifies a retrained method, and superscripts ^1^ and ^2^ identify results reported in [19,41], respectively. Metric abbreviations are as follows: Abs Rel, absolute relative error; Sq Rel, squared relative error; RMSE, root mean square error; ${\mathrm{RMSE}}_{\log}$, logarithmic root mean square error; and $\delta_{1}$–$\delta_{3}$, threshold accuracies.

Method	Max Depth	Depth Error (↓)				Depth Accuracy (↑)		
		**Abs Rel**	**Sq Rel**	**RMSE**	${\mathrm{RMSE}}_{\log}$	$\delta_{1}$	$\delta_{2}$	$\delta_{3}$
Monodepth2 * [15]	40 m	0.470	8.799	11.431	0.493	0.515	0.767	0.895
SwinDepth * [16]	40 m	0.372	6.251	10.692	0.427	0.534	0.811	0.926
Lite-Mono * [44]	40 m	0.385	4.610	11.911	0.435	0.390	0.686	0.867
ADFA ^2^ [21]	40 m	0.201	2.575	7.172	0.278	0.735	0.883	0.942
ADDS * [39]	40 m	0.239	1.455	4.979	0.288	0.579	0.889	0.961
RNW * [24]	40 m	0.178	1.474	5.256	0.226	0.752	0.919	0.974
STEPS * [25]	40 m	0.165	1.071	4.402	0.220	0.764	0.937	0.981
SRNSD ^2^ [41]	40 m	0.136	0.799	4.257	0.194	0.836	0.951	0.983
**Ours**	40 m	**0.111**	**0.661**	**3.541**	**0.168**	**0.887**	**0.963**	**0.986**
Monodepth2 * [15]	60 m	0.502	11.699	11.620	0.503	0.530	0.778	0.894
SwinDepth * [16]	60 m	0.396	8.580	10.663	0.435	0.554	0.826	0.927
Lite-Mono * [44]	60 m	0.393	4.954	12.004	0.438	0.393	0.689	0.863
ADFA ^1^ [21]	60 m	0.233	3.783	10.089	0.319	0.668	0.884	0.924
ADDS * [39]	60 m	0.248	1.729	5.434	0.293	0.567	0.880	0.957
RNW * [24]	60 m	0.213	2.975	7.590	0.262	0.729	0.890	0.952
GlocalFuse-Depth ^1^ [19]	60 m	0.217	2.299	8.520	0.261	0.672	0.901	0.961
STEPS * [25]	60 m	0.176	1.398	5.039	0.229	0.745	0.929	0.977
SRNSD ^2^ [41]	60 m	0.169	1.450	6.439	0.226	0.768	0.926	0.975
**Ours**	60 m	**0.116**	**0.850**	**3.962**	**0.174**	**0.876**	**0.958**	**0.984**

**Table 2:** Quantitative comparison on nuScenes-Night at maximum depths of 40 and 60 m. Arrows indicate the preferred metric direction. ↓ denotes that lower values are better, whereas ↑ denotes that higher values are better. Bold marks the best result at each depth cap, and an asterisk identifies a retrained method.

Method	Max Depth	Depth Error (↓)				Depth Accuracy (↑)		
		**Abs Rel**	**Sq Rel**	**RMSE**	${\mathrm{RMSE}}_{\log}$	$\delta_{1}$	$\delta_{2}$	$\delta_{3}$
Monodepth2 * [15]	40 m	0.554	10.132	10.234	0.581	0.431	0.672	0.796
SwinDepth * [16]	40 m	0.528	11.962	10.875	0.548	0.420	0.681	0.822
Lite-Mono * [44]	40 m	0.532	9.067	9.815	0.567	0.442	0.683	0.805
RNW * [24]	40 m	0.281	3.090	6.699	0.361	0.633	0.846	0.922
STEPS * [25]	40 m	0.268	2.708	6.318	0.349	0.629	0.855	0.927
**Ours**	40 m	**0.242**	**1.890**	**5.474**	**0.314**	**0.662**	**0.876**	**0.945**
Monodepth2 * [15]	60 m	0.549	10.792	13.677	0.646	0.392	0.621	0.752
SwinDepth * [16]	60 m	0.528	12.546	14.087	0.606	0.382	0.630	0.777
Lite-Mono * [44]	60 m	0.529	9.849	13.299	0.632	0.402	0.630	0.761
RNW * [24]	60 m	0.347	6.138	11.287	0.431	0.576	0.783	0.883
STEPS * [25]	60 m	0.315	4.790	10.173	0.490	0.571	0.801	0.897
**Ours**	60 m	**0.277**	**3.140**	**8.904**	**0.368**	**0.589**	**0.818**	**0.913**

**Table 3:** Cross-domain evaluation at 60 m after training on RobotCar-Night and testing directly on nuScenes-Night without target-domain fine-tuning. Arrows indicate the preferred metric direction. ↓ denotes that lower values are better, whereas ↑ denotes that higher values are better. Bold marks the best result, and an asterisk identifies a retrained method. Superscript ^1^ identifies results reported in [41].

Method	Max Depth	Depth Error (↓)				Depth Accuracy (↑)		
		**Abs Rel**	**Sq Rel**	**RMSE**	${\mathrm{RMSE}}_{\log}$	$\delta_{1}$	$\delta_{2}$	$\delta_{3}$
Monodepth2 * [15]	60 m	0.472	6.713	14.852	0.641	0.331	0.568	0.716
Lite-Mono * [44]	60 m	0.494	7.981	14.735	0.622	0.364	0.604	0.740
SwinDepth * [16]	60 m	0.403	5.802	14.101	0.562	0.398	0.647	0.781
ADDS * [39]	60 m	0.520	6.954	15.030	0.657	0.253	0.507	0.714
RNW * [24]	60 m	0.412	6.111	12.236	0.561	0.448	0.677	0.799
STEPS * [25]	60 m	0.417	6.654	12.804	0.563	0.444	0.668	0.794
SRNSD ^1^ [41]	60 m	0.352	4.420	11.942	0.515	0.419	0.650	0.790
**Ours**	60 m	**0.297**	**3.379**	**10.024**	**0.424**	**0.541**	**0.748**	**0.869**

**Table 4:** Component-level ablation of DIDAF-Depth on RobotCar-Night at 60 m. A ✓ indicates module inclusion; TC denotes the parallel Transformer-CNN encoder without VIF. Params count the depth network only. When the TC-VIF encoder is not used, the corresponding baseline encoder is adopted, namely, ResNet50 for EDCMN only (24.915 M) or TC for TC + DCAFM (47.337 M); when EDCMN is not used, the Monodepth2 decoder is adopted (8.289 M). ↓ and ↑ indicate that lower and higher values are better, respectively, and bold marks the best result.

Method	Params (M)	TC-VIF Encoder (49.151 M)	DCAFM (6.686 M)	EDCMN (1.118 M)	Depth Error (↓)				Depth Accuracy (↑)		
					**Abs Rel**	**Sq Rel**	**RMSE**	${\mathrm{RMSE}}_{\log}$	$\delta_{1}$	$\delta_{2}$	$\delta_{3}$
EDCMN only	26.033			✓	0.160	1.344	5.358	0.214	0.818	0.938	0.978
TC+DCAFM	62.312		✓		0.142	1.148	4.968	0.199	0.819	0.944	0.981
TC-VIF encoder only	57.440	✓			0.146	1.021	4.524	0.200	0.832	0.946	0.979
TC-VIF encode + EDCMN	50.268	✓		✓	0.140	0.880	4.265	0.196	0.836	0.952	0.983
TC-VIF encoder + DCAFM	64.126	✓	✓		0.134	0.956	4.508	0.189	0.842	0.951	0.983
Full DIDAF-Depth	56.954	✓	✓	✓	**0.116**	**0.850**	**3.962**	**0.174**	**0.876**	**0.958**	**0.984**

**Table 5:** Encoder ablation on RobotCar-Night at 60 m. TC denotes the parallel Transformer-CNN encoder without VIF; superscripts ^a–c^ refer to the architectures in Figure 2a–c. ↓ and ↑ indicate that lower and higher values are better, respectively, and bold marks the best result.

Method	Depth Error (↓)				Depth Accuracy (↑)		
	**Abs Rel**	**Sq Rel**	**RMSE**	${\mathrm{RMSE}}_{\log}$	$\delta_{1}$	$\delta_{2}$	$\delta_{3}$
CNN only	0.176	1.398	5.039	0.229	0.745	0.929	0.977
Transformer only	0.162	1.573	5.626	0.218	0.797	0.927	0.972
TC only	0.230	3.076	7.700	0.307	0.700	0.869	0.936
Sequential stacking ^a^ [17]	0.200	1.674	5.963	0.247	0.711	0.916	0.975
Integrated mixing ^b^ [18]	0.157	1.642	5.979	0.223	0.804	0.931	0.936
Parallel branches ^c^ [19]	0.154	1.370	5.274	0.208	0.809	0.936	0.970
TC-VIF encoder	**0.146**	**1.021**	**4.524**	**0.200**	**0.832**	**0.946**	**0.979**

**Table 6:** Ablation of DCAFM attention and fusion strategies on RobotCar-Night at 60 m. In the row labels, C and T denote CNN and Transformer features, respectively; *w*/*o* denotes without. ↓ and ↑ indicate that lower and higher values are better, respectively, and bold marks the best result.

Method	Depth Error (↓)				Depth Accuracy (↑)		
	**Abs Rel**	**Sq Rel**	**RMSE**	${\mathrm{RMSE}}_{\log}$	$\delta_{1}$	$\delta_{2}$	$\delta_{3}$
Channel attention only	0.151	0.918	4.274	0.206	0.814	0.951	0.981
Spatial attention only	0.146	0.975	4.509	0.212	0.805	0.945	0.978
Channel (C) + Spatial (T)	0.139	0.971	4.453	0.196	0.839	0.948	0.980
Channel (T) + Spatial (C)	0.141	1.126	4.949	0.200	0.826	0.944	0.980
DCAFM *w*/*o* residual refinement	0.152	1.071	4.764	0.206	0.808	0.951	0.982
Feature-map addition	0.133	**0.792**	4.126	0.192	0.828	0.952	**0.984**
GlocalFuse-Depth fusion [19]	0.130	0.981	4.496	0.188	0.850	0.950	0.981
DCAFM	**0.116**	0.850	**3.962**	**0.174**	**0.876**	**0.958**	**0.984**

**Table 7:** Decoder-component ablation on RobotCar-Night at 60 m. EUCB, FPN, PPM, and DC identify the removed EDCMN components; *w*/*o* denotes without. ↓ and ↑ indicate that lower and higher values are better, respectively, and bold marks the best result.

Method	Depth Error (↓)				Depth Accuracy (↑)		
	**Abs Rel**	**Sq Rel**	**RMSE**	${\mathrm{RMSE}}_{\log}$	$\delta_{1}$	$\delta_{2}$	$\delta_{3}$
EDCMN *w*/*o* EUCB	0.133	1.004	4.589	0.191	0.832	0.948	0.982
EDCMN *w*/*o* FPN	0.150	1.230	5.123	0.207	0.813	0.941	0.978
EDCMN *w*/*o* PPM	0.145	1.252	5.130	0.200	0.827	0.940	0.979
EDCMN *w*/*o* DC	0.136	0.880	4.207	0.186	0.840	0.952	**0.984**
Full EDCMN	**0.116**	**0.850**	**3.962**	**0.174**	**0.876**	**0.958**	**0.984**

## Data Availability

The Oxford RobotCar dataset (https://robotcar-dataset.robots.ox.ac.uk/ (accessed on 3 August 2026)) and nuScenes dataset (https://www.nuscenes.org/nuscenes (accessed on 3 August 2026)) used in this study are publicly available from their official websites. No new raw driving dataset was generated. The dataset construction, preprocessing procedures, and train/test split definitions used in the experiments are described in the Datasets section of this article. The source code and trained model weights supporting this study are available from the corresponding author upon reasonable request.
